# Adhesion of Soft Palate to Pharynx, Operation ; Cure

**Published:** 1891-03

**Authors:** Hal Foster

**Affiliations:** Kansas City, Mo. Larynologist to All Saints' and Missouri Pacific R. R. Hospital.


ARTICLE V.

### ADHESION OF SOFT PALATE TO PHARYNX; OPERATION; CURE.

BY HAL FOSTER, M. D., KANSAS CITY, MO.

Larynologist to All Saints' and Missouri Pacific R. R. Hospital.

Read before the Medical Society of the Missouri Valley, December, 1890.

The patient, a man 40 years old, by occupation a seaman, and a native of France, applied to me November, 1889, complaining of shortness of breath. He wanted to know if anything could be done to restore his speech. He was able to utter a sound, but he could not turn this sound into words.

He had contracted syphilis twenty years before. He was married, and his wife had miscarried three times. One child was born living, and is now twelve years of age, and apparently in fair health. This communication was made on paper, as the patient was unable to talk. His nose was entirely eaten away down to the nasal bones, so that all that remained was a small opening in the face. The nasal fossæ would fill up with large green crusts, which he dislodged with a hair-pin every morning and evening. The stench from these crusts was exceedingly offensive. I took out two, which were fully ten and a half inches in length, and shell-like in form. The patient's mouth was so closed up by syphilitic adhesion that I could pass in my two fingers only with great difficulty, by stretching the mouth to its utmost capacity. A laryngoscopic view of the throat at this stage was impossible.

The first operation I undertook was enlargement of the mouth, which was done by means of a knife. Afterwards little wooden stretchers or gags were inserted, to prevent the wounded surfaces from growing together again. After this was accomplished, I found that the uvula and the lower part of the soft palate were entirely destroyed, and the remaining part of the palate was firmly adherent to the pharynx, so that the upper pharynx was completely separate from the lower pharynx. The complete occlusion of the two pharynges is extremely rare. I have met with it in only three cases. It is by no means rare in tertiary syphilis of the throat to find a partial adhesion of the parts; but it is extremely rare to find a complete adhesion. Often a small opening can be discovered, sometimes only large enough to admit a small probe, but still large enough to admit a small current of air.

In this case I covered the lower pharynx with iodoform and filled the nasal fossæ with water. My iodoform was not moistened.

I now determined to liberate the palate from the pharynx, so that I could give my patient nasal breathing, and also give the nasal cavity an outlet into the throat. I first passed a silver probe down through the nose to the pharynx, and with a tenotomy knife cut where the palate bellied. After I had thoroughly separated the palate from its attachment, with no little difficulty, the parts were sprayed with a solution of tannin. No anæsthetic of any kind was used. I now began to pass the largest sound I could obtain through the opening into the pharynx. I afterwards had a sound made after the following description. The body of the instrument was about two inches in length, and it was blunt-pointed at the end, and gradually as it reached the base, it was somewhat similar to a triangle in shape. The base was an inch and a half in width. To the broad end of the instrument was attached a long handle, so that it could be manipulated in the throat. I found that with this instrument I could keep the opening the required size and prevent it from closing. Had the wound not been treated with sounds after cutting, the opening would have filled up in a very short time.

This is a peculiar characteristic of syphilitic tissue, where we seek to enlarge an opening, whether it be in the pharynx or nasal fossæ. In a case of nasal stenosis now under observation, I have seen the parts close completely after opening the nasal canal three times, every time giving the patient complete breathing space, taking out at each operation a large piece of cartilage at the point of union. In a short time the parts were firmly adherent again.

The parts in this operation healed nicely, and within four days absolutely no pain was felt from the passage of the instrument. An examination of the larynx showed complete paralysis of the abductors of the vocal cords, and the whole mucous membrane lining the larynx was in a state of hyperemia. This accounted for the loss of voice. At the beginning of my operations, I had placed my patient under iodide of potassium; but I was compelled to give him 90 grains, three times a day, before I could produce any effect. I do not consider this an unusually large dose in cases of tertiary syphilis of the throat and nose, and have often given larger doses and maintained them during a longer time. This was given in Vichy water; his stomach stood it nicely, and I kept this amount up for two weeks, when it was gradually reduced. Later I gave him only tonic doses. Within a month and a half from the time of the first operation on the mouth, the patient had entirely regained his voice, and had gained something like fifteen pounds in weight. His voice was now quite clear and strong. I had an artificial nose made for him by

Tiemann & Co., while I was attending the Manhattan Throat Hospital of New York last August, which supplied his missing member very well. I gave him one of the sounds above described, and told him to use it once or twice a week for a year. I often hear from him, and he reports the breathing through his artificial member excellent, and his voice good.—*Med. and Surg. Reporter.*

